# Incontinence of Urine Cured by Hepatised Ammonia

**Published:** 1799-10

**Authors:** 


### *To the Editors of the Medical and Phyſical Journal.*

Gentlemen,

From the variety of new remedies lately introduced into the Medical World, and the conſequent inquiries into their reſpective efficacies, made by Profeſſors, I conceive that the ſuccefsful application of any one of them, (as on the other hand its failure) ſhould be communicated publicly and undiſguiſedly, by which means we ſhall be more certainly able to aſcertain the advantages likely to reſult from their adoption and adminiſtration. Impreſſed with this idea, I venture to trouble you with a caſe in which great benefit ſeems to have been derived from the uſe of the *hepatiſed ammonia.*

A young man with whom I am acquainted, had been, from his infancy, troubled with an incontinence of urine, the diſcharge of which he was not at any time able to ſupprefs, particularly during night. The copious evacuation of this ſecretion neceſſarily cauſed a conſtant and conſiderable degree of debility, but I never noticed any ſymptoms of hectic. A diſeaſe ſo diſtreſſing and unpleaſant in itſelf, naturally induced his friends to ſeek every poſſible means of relief, by applying to ſeveral phyſicians of eminence, whoſe preſcriptions and advice, although exactly followed, produced no good effect; on the contrary, the malady continued to increaſe with his years. I had heard much promiſed from the introduction of the hepatiſed ammonia, and was tempted in this inſtance to eſſay its virtue. I previouſly examined the ſtate and appearance of the urine voided, and found it to poſſeſs both that peculiar ſmell and ſaccharine taſte, ſo commonly diſtinguiſhed in caſes of diabetes. On holding it to the light in a glaſs veſſel, veſſel, it exhibited the appearance of a bluiſh-red colour, which rendered it ſomewhat cloudy and opaque; placed in any other ſituation, it appeared perfectly limpid. He began according to my directions, with taking three drops of the ſpecific, night and morning in a little water; this he gradually increaſed to twenty or twenty-five drops each doſe: I alſo deſired him to uſe animal, and abſtain from vegetable food, and ordered for his common drink ſome water of an alkaline quality. In this regimen he punctually perſiſted, till he obtained the intended benefit: from the commencement of this courſe, he gradually amended, the evacuations of his urine became leſs frequent, and at length not involuntary; by degrees it perceptibly loſt its unhealthy taſte, ſmell, and colour, and in ſhort, he is at this time entirely releaſed from his diſagreeable diſorder, and ſeems to acquire daily his uſual ſtrength and vigour.

Should this ſimple relation afford ſatisfaction to any of your numerous readers, I ſhall feel myſelf favoured by its inſertion in your very intereſting Miſcellany.

I am, Gentlemen,

Bradford, Wilts, With reſpect,

*Sept.* 11, 1799. Your obedient ſervant,

